# *Helicobacter pylori* Vacuolating Cytotoxin A Causes Anorexia and Anxiety via Hypothalamic Urocortin 1 in Mice

**DOI:** 10.1038/s41598-019-42163-4

**Published:** 2019-04-12

**Authors:** Hajime Suzuki, Koji Ataka, Akihiro Asakawa, Kai-Chun Cheng, Miharu Ushikai, Haruki Iwai, Takakazu Yagi, Takeshi Arai, Kinnosuke Yahiro, Katsuhiro Yamamoto, Yoshito Yokoyama, Masayasu Kojima, Toshihiko Yada, Toshiya Hirayama, Norifumi Nakamura, Akio Inui

**Affiliations:** 1 Department of Oral and Maxillofacial Surgery, Kagoshima University Graduate School of Medical and Dental Sciences, Kagoshima, Japan; 2 Department of Psychosomatic Internal Medicine, Kagoshima University Graduate School of Medical and Dental Sciences, Kagoshima, Japan; 3 Department of Pharmacological Sciences of Herbal Medicine, Kagoshima University Graduate School of Medical and Dental Sciences, Kagoshima, Japan; 4 Department of Hygiene and Health Promotion Medicine, Kagoshima University Graduate School of Medical and Dental Sciences, Kagoshima, Japan; 5 Department of Oral Anatomy and Cell Biology, Kagoshima University Graduate School of Medical and Dental Sciences, Kagoshima, Japan; 6 Department of Orthodontics and Dentofacial Orthopedics, Kagoshima University Graduate School of Medical and Dental Sciences, Kagoshima, Japan; 7 Department of Physiology, Jichi Medical University School of Medicine, Tochigi, Japan; 8 Department of Molecular Infectiology, Graduate School of Medicine, Chiba University, Chiba, Japan; 9 Quality Control Department, Yoshitomi Plant, Mitsubishi Tanabe Pharma Factory Ltd., Fukuoka, Japan; 10 Discovery Technology Laboratories, Sohyaku. Innovative Research Division, Mitsubishi Tanabe Pharma Corporation, Saitama, Japan; 11 Molecular Genetics, Institute of Life Science, Kurume University, Fukuoka, Japan; 12 Department of Bacteriology, Institute of Tropical Medicine, Nagasaki University, Nagasaki, Japan

## Abstract

*Helicobacter pylori* (*Hp*) infection is related to the pathogenesis of chronic gastric disorders and extragastric diseases. Here, we examined the anorexigenic and anxiogenic effects of *Hp* vacuolating cytotoxin A (VacA) through activation of hypothalamic urocortin1 (Ucn1). VacA was detected in the hypothalamus after peripheral administration and increased Ucn1 mRNA expression and c-Fos-positive cells in the hypothalamus but not in the nucleus tractus solitarius. c-Fos and Ucn1-double positive cells were detected. CRF1 and CRF2 receptor antagonists suppressed VacA-induced anxiety and anorexia, respectively. VacA activated single paraventricular nucleus neurons and A7r5 cells; this activation was inhibited by phospholipase C (PLC) and protein kinase C (PKC) inhibitors. VacA causes anorexia and anxiety through the intracellular PLC-PKC pathway, migrates across the blood-brain barrier, and activates the Ucn1-CRF receptor axis.

## Introduction

*Helicobacter pylori* (*Hp*) infection of humans is considered to have first occurred approximately 58,000 ± 3500 years ago in East Africa and has since spread worldwide with racial migrations^1^. Currently, more than 50% of the global population are carriers^2^. *Hp* infection is a major contributor to the pathogenesis of chronic gastric disorders, such as functional dyspepsia (FD), peptic ulcer disease, gastric adenocarcinoma, and mucosa-associated lymphoid tissue lymphoma^3^ and is also potentially associated with extragastric diseases, such as cardiovascular diseases, diabetes, hematological diseases, hepatobiliary diseases, and dementia^4^. The prevalence of *Hp* infection has an inverse correlation with obesity in European countries, Japan, the United States, and Australia^5^. Eradication therapy for *Hp*, which improves gastrointestinal symptoms and the risk of gastric cancer in FD patients with *Hp* infection^6^, is associated with body weight gain^7^. Furthermore, the appetite index measured with a visual analog scale (VAS) was higher in patients in whom eradication therapy was successful was than in those in whom it failed^8^. Appetite is modulated by peripheral hormones and central neuropeptides^9^. Ghrelin is the only peripheral orexigenic peptide produced in the stomach, and it shares a close relationship with the brain-gut axis^10^. *Hp* colonization does not alter the plasma ghrelin levels in specific-pathogen-free and germ-free mice^11^. Human studies have reported that the ghrelin levels are lower in *Hp*-positive patients than in *Hp*-negative patients, whereas other studies have shown that the plasma ghrelin levels are similar in *Hp*-positive and *Hp*-negative patients^12^. Similar conflicting results concerning circulating ghrelin levels have been reported in subjects who exhibited increases in body weight and food intake after *Hp* eradication^12^. Therefore, the relationship between ghrelin and *Hp*-induced decreases in body weight and appetite is limited and remains unclear.

When the brain receives stimuli from various stressors, intestinal functions change and induce various intestinal symptoms, such as diarrhea and visceral pain (brain-gut interaction). Additionally, many peptides are produced by intestines, such as ghrelin, cholecystokinin, and glucagon-like peptide-1 regulate appetite (gut-brain interaction)^9^. Thus, the gastrointestinal tract and central nervous system share a bidirectional neurohumoral communication that is defined as the brain-gut axis^13^. Various gastrointestinal disorders have been reported to increase comorbidity in psychiatric disorders^14,15^, and central neuromodulators, which are used to treat depression and anxiety, have also been used to treat gastrointestinal symptoms based on the gut-brain interaction^16^. The gut microbiota has recently been found to be a key player in the brain-gut axis^17^ and has been reported to share a relationship with neuropsychiatric disorders^18^. FD, a common gastrointestinal disease, can be caused by *Hp* infection and has been reported to be associated with higher scores for anxiety, depression, and psychological distress^19^. Recently, a cross-sectional study in humans reported that *Hp* infection was a risk factor for psychological distress and depressed mood^20^. However, the mechanism underlying this remain is unclear.

Vacuolating cytotoxin A (VacA) is a major virulence factor produced by almost all *Hp* strains that causes *Hp*-associated disorders^21^. VacA (90-kDa toxin protein) is activated in low *pH* environments, such as that found in the stomach^22^. The C-terminal region of VacA contains binding sites for toxin receptors on the cell membrane, such as the low-density lipoprotein receptor-related protein-1 (LRP1), which is expressed on gastric epithelial cells and the hypothalamus^23,24^. VacA induces the formation of large vacuoles in the cytoplasm, mitochondrial-dependent apoptosis and autophagy of epithelial cells, and the inhibition of T cell proliferation^21^. Both VacA and serum VacA antibodies are associated with an increased risk of gastroduodenal ulcers and gastric cancer^25,26^. However, an association of VacA with psychological disorders, such as anxiety and anorexia, has not been demonstrated to date.

The aims of this study were to confirm the anorexigenic and anxiogenic effects of *Hp* VacA and its mechanisms of action using animal models.

## Results

### Chronic *Hp* infection inhibits food intake and body weight gain in Mongolian gerbils

**Figure 1:**
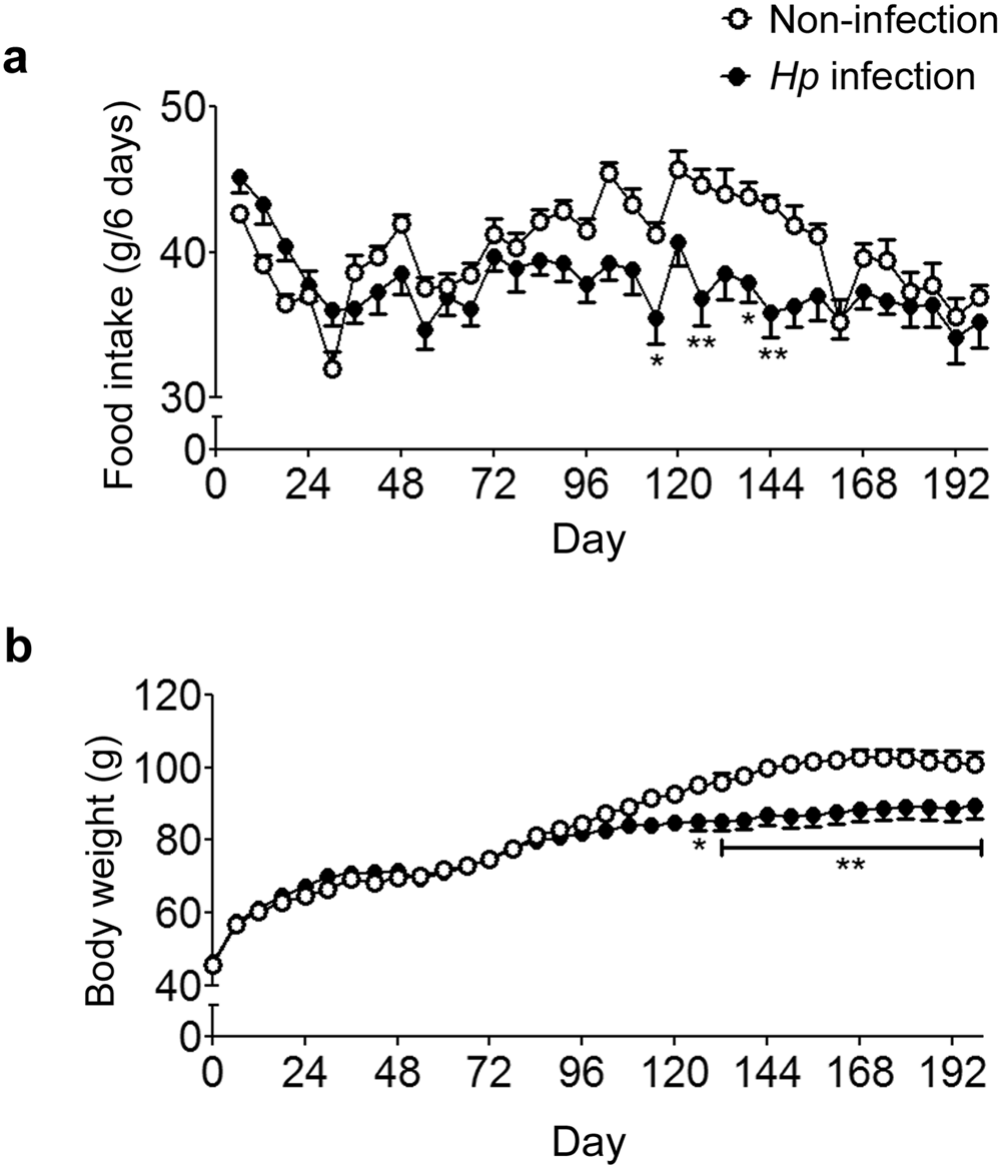
Chronic *Hp* infection in Mongolian gerbils inhibits food intake and body weight gain. (**a**,**b)** Cumulative food intake **(a)** and body weight **(b)** were measured for 198 days (*n* = 10). The values are presented as the means ± SEM. Differences were considered significant at *p < 0.05 or **p < 0.01 compared with the non-infected or vehicle-treated group.

Mongolian gerbils that were confirmed to have an *Hp* infection were included in the infection group and produced 46 ± 26 colonies, which was significantly higher than the number produced in the non-infection group (0 ± 0 colonies). *Hp* significantly suppressed the cumulative food intake at 102, 126, 138, 144, and 150 days post-infection (F_1, 18_ = 15.40, *p* = 0.0321, two-way ANOVA; Fig. 1a) and inhibited body weight gain from days 126 to 198 (F_1, 18_ = 7.08, *p* = 0.0159, two-way ANOVA; Fig. 1b).

### VacA induces anorexia, anxiety, and the mRNA expression of urocortin1 (Ucn1) in mice

**Figure 2:**
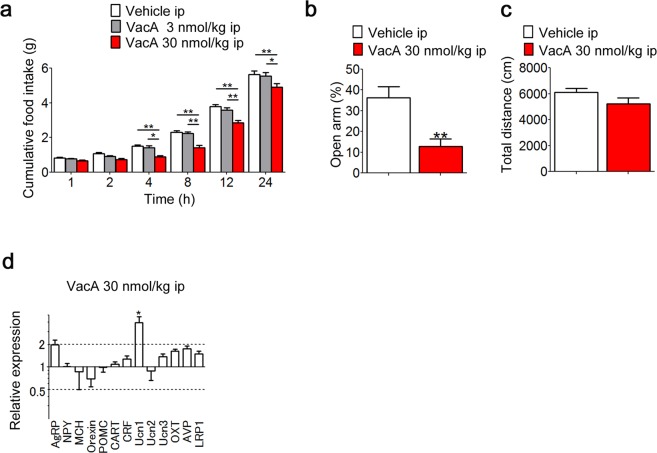
Peripheral VacA administration induces anorexia, anxiety, and the mRNA expression of Ucn1 in mice. (**a)** Cumulative food intake was measured for 24 h in mice receiving IP administration (*n* = 8–10). (**b**,**c**) The percentage of time spent in the open arms (**b**) and total distance (**c**) were measured 4 h after the IP administration of 30 nmol/kg bw of VacA in mice (*n* = 5–8). TBS (vehicle) was administered as the control. (**d**) Four hours after IP administration of 30 nmol/kg bw of VacA to mice, the orexigenic and anorexigenic peptide mRNA levels in the hypothalamus were measured by qRT-PCR (*n* = 5–8). The values are presented as the means ± SEM. Differences were considered significant at *p < 0.05 and **p < 0.01. AgRP, Agouti-related peptide; NPY, neuropeptide Y; MCH, melanin-concentrating hormone; POMC, proopiomelanocortin; CART, cocaine- and amphetamine-regulated transcript; CRF, corticotropin-releasing factor; Ucn, urocortin; OXT, oxytocin; AVP, arginine vasopressin; LRP1, low-density lipoprotein receptor-related protein-1.

Intraperitoneal (IP) administration of 30 nmol/kg of body weight (kg bw) VacA significantly decreased cumulative food intake from 4 to 24 h after administration to below what was found in the vehicle-administered control group (F_2, 32_ = 12.99, *p* < 0.0001, two-way ANOVA; Fig. 2a). There were no significant differences between the 3 nmol/kg bw of VacA group and the vehicle control group at any time (Fig. 2a). IP administration of VacA at the same dosage had an anorexigenic effect by which it decreased the percentage of time spent in the open arms of the elevated plus maze test (Fig. 2b). There were no significant differences between the 30 nmol/kg bw of VacA group and the vehicle control group in total distance of the elevated plus maze test (Fig. 2c). The delta delta CT (2^−ΔΔCT^) value for Ucn1 was 3.956 in the hypothalamus of mice subjected to IP administration of VacA, which was significantly higher than the level in the vehicle-administered control group (Fig. 2d). Following IP administration of VacA, the 2^−ΔΔCT^ values for agouti-related peptide (AgRP) was 1.982, indicating a difference of no more than 2-fold (Fig. 2d). The expression levels of the other mRNAs were not altered by VacA administration (Fig. 2d).

### VacA directly induces neuron activity in hypothalamus

**Figure 3:**
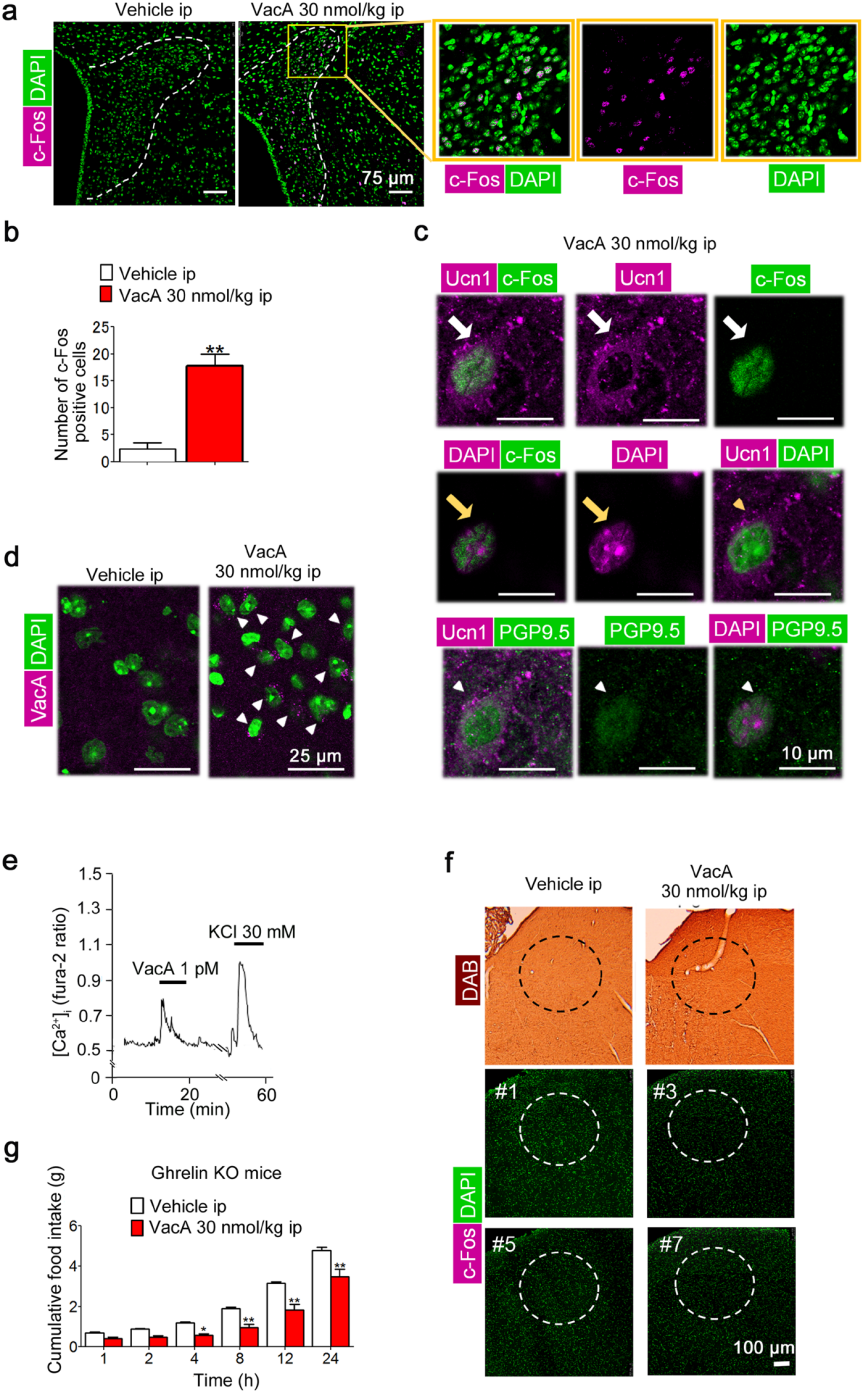
Peripheral VacA administration directly induces neuron activity in hypothalamus. Each brain was isolated and fixed with 4% PFA and 0.5% GA in 0.1 M PB 4 h after IP administration of 30 nmol/kg bw of VacA. Coronal sections of the PVN were stained with an anti-mouse c-Fos and/or Ucn1antibody. (**a**) Representative images of the c-Fos-positive cells in the PVN using immunofluorescent staining. (**b**) The number of c-Fos-positive cells in the PVN was counted on one side of the PVN (*n* = 4). (**c**) Representative images of c-Fos- and Ucn1-positive (white arrows), c-Fos- and DAPI-positive (yellow arrows), Ucn1- and DAPI-positive cells (yellow arrow heads), or Ucn1- and PGP9.5-positive cells (white arrow heads) in the PVN cells. (**d**) Representative images in the hypothalamus obtained after immunofluorescent staining with VacA antibody (arrows). (**e)** Representative [Ca^2+^]i oscillations (Fura-2 ratio) in single cells administered 1 pM of VacA. (**f)** Representative images of c-Fos-positive cells in the NTS obtained after DAB or immunofluorescent staining (# number indicates an individual animal) are shown. (**g**) Cumulative food intake was measured for 24 h in ghrelin-KO mice receiving IP VacA administration. TBS was administered as the control. The values are presented as the means ± SEM. Differences were considered significant at *p < 0.05 and **p < 0.01.

IP administration of 30 nmol/kg bw VacA induced an increase in the number of c-Fos-positive cells in the paraventricular nucleus (PVN) of the hypothalamus (Fig. 3a,b). c-Fos-positive cells were also Ucn1-positive (Fig. 3c). Double immunofluorescence for Ucn1 and the neuronal marker protein gene product 9.5 (PGP 9.5) was also performed to investigate whether Ucn1 existed in neuronal cells. Ucn1-positive cells were PGP9.5-positive cells (the representative image was shown in Fig. 3c). VacA was detected in the hypothalamus (Fig. 3d). We then investigated whether VacA directly activated PVN neurons by measuring the cytosolic Ca^2+^ concentration ([Ca^2+^]_i_). VacA administered at a dose of 1 pM increased the [Ca^2+^]_i_ of single PVN neurons (Fig. 3e). Among the 208 single neurons we observed, 17 single neurons (8.17%) were activated by VacA. c-Fos-positive cells were not detected in the nucleus tractus solitarius (NTS) based on 3,3′-diaminobenzidine (DAB) staining (Fig. 3f) and immunofluorescence (Fig. 3g). Ghrelin-knockout (KO) mice were IP administered the same effective doses of VacA used in the wild-type mice to examine whether ghrelin participated in the action mechanisms of VacA. The administration of 30 nmol/kg bw of VacA significantly decreased cumulative food intake from 4 to 24 h post-administration (F_1, 16_ = 23.97, *p* = 0.0002, two-way ANOVA; Fig. 3g).

### VacA induces anorexia and anxiety via corticotropin-releasing factor (CRF) receptors

**Figure 4:**
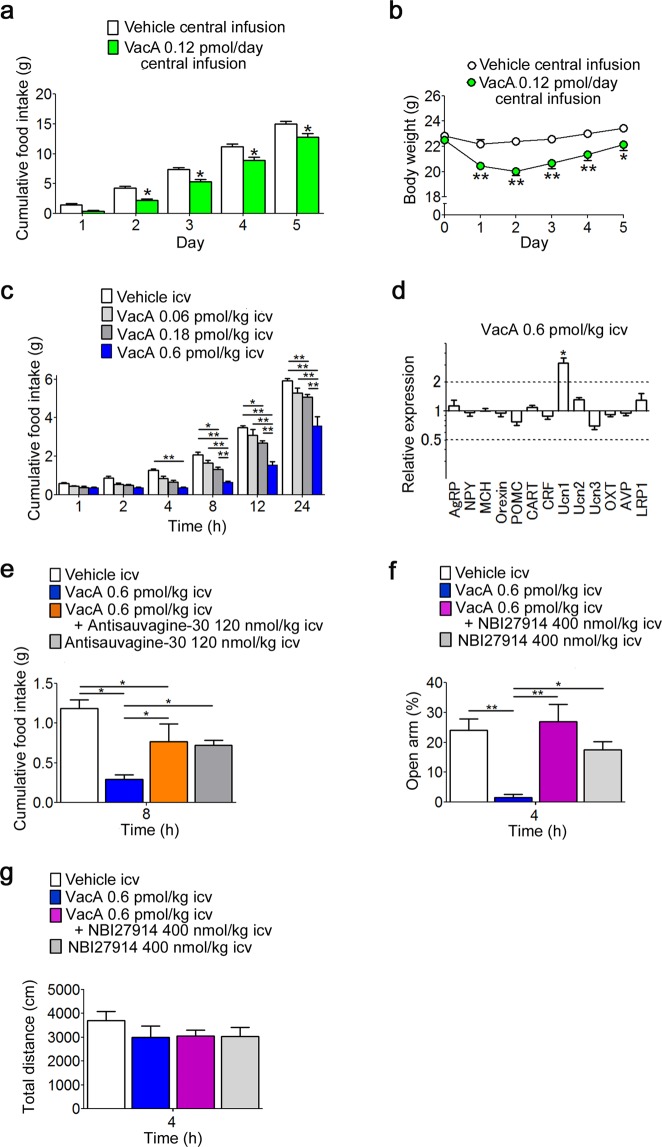
Central VacA administration induces anorexia and anxiety via CRF receptors. **(a**,**b)** Cumulative food intake **(a)** and body weight **(b)** were measured for 5 days in mice that received a central subchronic infusion of VacA (*n* = 8). (**c**) Cumulative food intake was measured for 24 h in mice receiving ICV administration (*n* = 9). (**d**) Four hours after ICV administration of 0.6 pmol/kg bw of VacA in mice, orexigenic and anorexigenic peptide mRNA levels were measured in the hypothalamus (*n* = 10–11). ACSF was administered as the vehicle control. (**e**) Cumulative food intake was measured for 8 h in mice deprived of food overnight and treated with an ICV administration of 0.6 pmol/kg bw of VacA or vehicle followed by ICV administration of 120 nmol/kg bw of the CRF2 antagonist antisauvagine-30 (*n* = 6–9). (**f**,**g**) The percentage of time spent in the open arms (**f**) and total distance (**g**) were measured in mice 4 h after ICV administration of 0.6 pmol/kg bw of VacA or vehicle followed by ICV administration of 400 nmol/kg bw of the CRF1 antagonist NBI27914 (*n* = 5). The values are presented as the means ± SEM. Differences were considered significant at *p < 0.05 or **p < 0.01. AgRP, Agouti-related peptide; NPY, neuropeptide Y; MCH, melanin-concentrating hormone; POMC, proopiomelanocortin; CART, cocaine- and amphetamine-regulated transcript; CRF, corticotropin-releasing factor; Ucn, urocortin; OXT, oxytocin; AVP, arginine vasopressin; LRP1, low-density lipoprotein receptor-related protein-1.

Because peripheral VacA seemed to directly alter neuron activity in the hypothalamus, we next tried to evaluate the effects of intracerebroventricular (ICV) administration of VacA on food intake, body weight, and anxiety-like behavior. The dosages used for the ICV administration of VacA were estimated from the effective concentration in single neurons. Subchronic central infusion of 0.12 pmol/day of VacA significantly suppressed cumulative food intake from days 2 to 5 (F_1, 21_ = 15.58, *p* = 0.0007, two-way ANOVA; Fig. 4a) and body weight gain from days 1 to 5 (F_1, 21_ = 18.6, *p* = 0.0003, two-way ANOVA; Fig. 4b). ICV administration of 0.6 pmol/kg bw of VacA significantly decreased cumulative food intake from 4 to 24 h, and 0.18 pmol/kg bw of VacA significantly decreased cumulative food intake from 8 to 24 h post-administration (F_3, 33_ = 22.02, *p* < 0.0001, two-way ANOVA; Fig. 4c). There were no significant differences between the 0.06 pmol/kg bw of VacA group and vehicle control group (Fig. 4c). We subsequently measured the mRNA expression levels of hypothalamic neuropeptides 4 h after the ICV administration of 0.6 pmol/kg bw of VacA in fasted mice using the 2^−ΔΔCT^ method. The value of Ucn1 in mice administered VacA via the ICV route was 3.148 and was significantly higher than the level in the vehicle-administered control group (Fig. 4d). The expression levels of the other mRNAs were not altered by VacA administration (Fig. 4d). We next examined whether CRF receptors, which are also Ucn1 receptors, participated in the mechanisms of action of VacA. ICV administration of the selective CRF2 antagonist antisauvagine-30 significantly inhibited the suppression of food intake induced by the ICV administration of VacA but did not recover it to the levels observed in control mice that received ICV administration of the vehicle (F_3,23_ = 13.25, *p* < 0.0001, two-way ANOVA; Fig. 4e). The ICV administration of VacA at the same dosage that showed an anorexigenic effect decreased the percentage of time spent in the open arms, whereas the ICV administration of the CRF1 antagonist NBI27914 significantly reversed the decrease in the percentage of time spent in the open arms induced by ICV administration of VacA to the level observed in the control mice that received ICV administration of the vehicle (F_3,16_ = 9.329, *p* = 0.0378, two-way ANOVA; Fig. 4f). There were no significant differences among each group in total distance of the elevated plus maze test (Fig. 4g).

### VacA activates the intracellular signals *in vitro*

**Figure 5:**
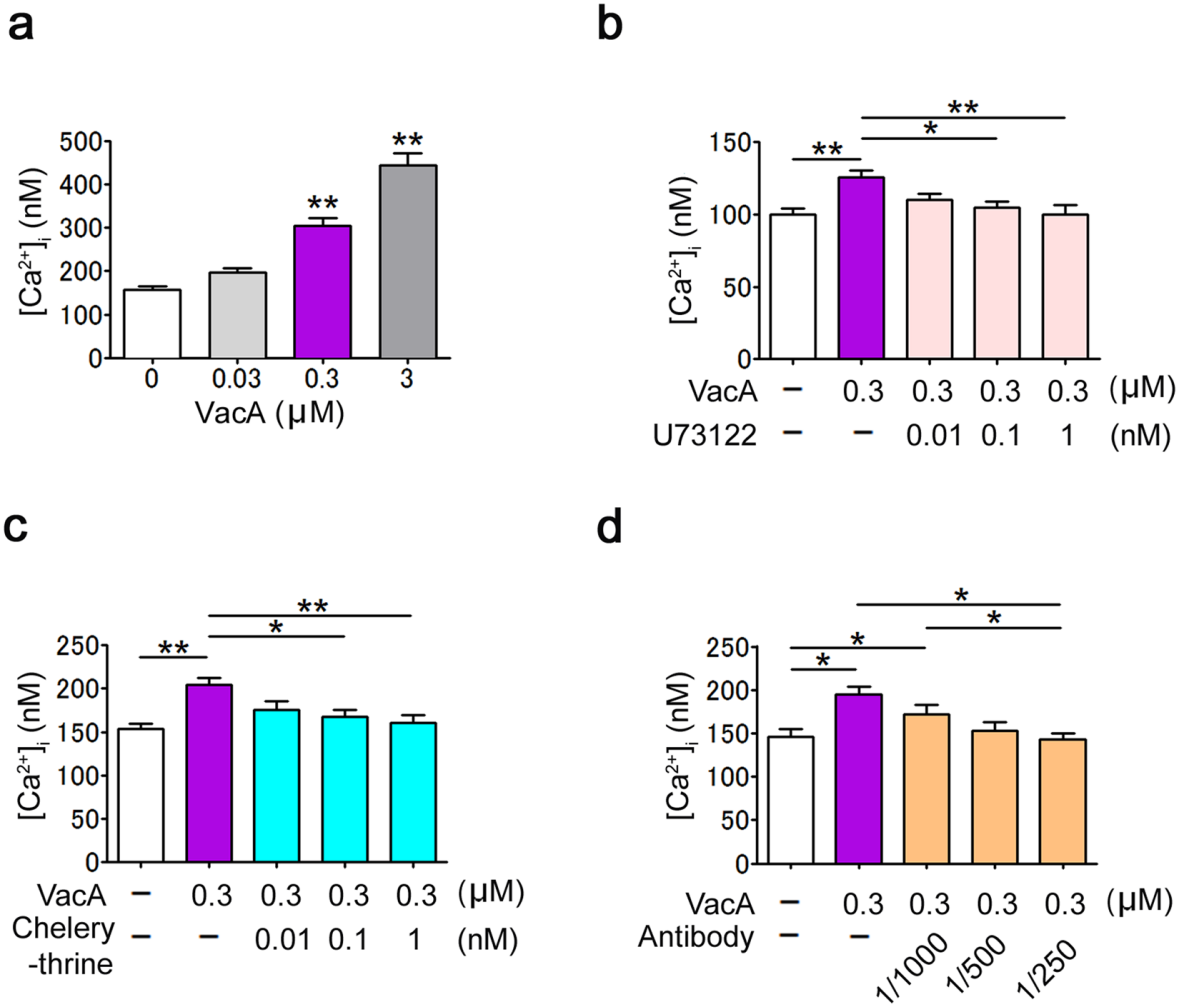
VacA increases [Ca^2+^]_i_ in A7r5 cells. **(a)** [Ca^2+^]_i_ was measured in A7r5 cells treated with 0, 0.03, 0.3, or 3 µM of VacA with vehicle (*n* = 6). (**b**–**d**) [Ca^2+^]_i_ was measured in A7r5 cells treated with 0.3 µM of VacA with vehicle, 0.01, 0.1, and 1 nM of the PLC inhibitor U73122 (*n* = 6, **b**); 0.01, 0.1, and 1 nM of the PKC inhibitor chelerythrine (*n* = 6, **c**); or 1:1000, 1:500 and 1:250 dilutions of an anti-VacA antibody (*n* = 6, **d**). The values are presented as the means ± SEM. Differences were considered significant at *p < 0.05 or **p < 0.01.

The effect of VacA on intracellular signaling was examined by determining the intracellular calcium concentration in A7r5 cells. VacA significantly increased the [Ca^2+^]_i_ in the A7r5 cells in a dose-dependent manner (F_3, 20_ = 50.80, *p* = 0.0043, one-way ANOVA; Fig. 5a). VacA-induced increases of [Ca^2+^]_i_ were inhibited by 0.1 and 1 nM of U-73122, a PLC inhibitor, (F_4, 25_ = 4.992, *p* < 0.0001, one-way ANOVA; Fig. 5b) and 0.1 and 1 nM of chelerythrine, a PKC inhibitor (F_4, 25_ = 6.052, *p* = 0.0015, one-way ANOVA; Fig. 5c). VacA-mediated increases of [Ca^2+^]_i_ were significantly suppressed in the presence of anti-VacA antibodies (1:250) (F_5,28_ = 5.036, *p* = 0.0020, one-way ANOVA; Fig. 5d).

## Discussion

Previous studies show that *Hp* infection has an association with body weight status^27–29^. Regarding appetite, *Hp* eradication has been reported to increase hunger scores assessed using a VAS^8^ and improve quality of life concerning eating habits in patients with peptic ulcer disease^30^. Our present study demonstrated that chronic *Hp* infection decreased both body weight and food intake in an animal model. These results strongly support the influence of *Hp* infection on the regulation of body weight and food intake.

Depression and anxiety are risk factors for functional gastrointestinal disorders, and environmental stressors alter the function of the gastrointestinal tract and symptoms in patients with functional gastrointestinal disorders^19,31^. The association between gastrointestinal disorders and anxiety has been well documented; however, little is known about the anxiogenic effect of *Hp*, which is considered an important risk factor for and cause of functional gastrointestinal disorders^2,3^. It was recently reported that *Hp* infection status is a risk factor for mental illness and depressed mood, and higher degrees of *Hp*-associated atrophic gastritis showed the highest risk of psychological distress and depression, although the mechanism remains to be elucidated^20^. The association between anxiety and appetite loss is also recognized^32^. In the present study, peripheral administration of VacA caused not only food intake loss but also anxiety in experimental animals. These results provide positive evidence that *Hp* causes anxiety and suggest that VacA is a cause of psychological symptoms, such as anxiety and appetite loss, in patients with *Hp* infection.

In the present study, both the peripheral and central administration of VacA decreased cumulative food intake and induced anxiety-like behaviors. When VacA was administered peripherally, the number of c-Fos-positive cells did not increase in the NTS, which is the relay region between the peripheral and central nervous systems. Furthermore, VacA was detected in the hypothalamus of mice with peripheral VacA administration. These results suggest that VacA exerts its pathological effects predominantly through the direct activation of cells in the hypothalamus, especially neurons in the PVN of the hypothalamus. VacA might affect the central nervous system as a humoral (endocrine) signal but not as an afferent neuronal signal.

Appetite is regulated by peripheral hormones and central neuropeptides. The hypothalamus, which includes the arcuate nucleus (ARC), PVN, lateral hypothalamus, and ventromedial hypothalamic nuclei, is the pivotal brain region that regulates appetite^9^. The PVN receives many projections from various brain regions, including orexigenic neuropeptide Y (NPY)/AgRP and anorexigenic proopiomelanocortin/α-melanocyte-stimulating hormone neurons in the ARC; therefore, the PVN is thought to coordinate feeding behavior^33^. Peripheral administration of VacA increased Ucn1 mRNA expression but did not alter the expression levels of other mRNAs for anorexigenic and orexigenic peptides in the hypothalamus in the present study. Peripheral administration of VacA increased the number of c-Fos-positive cells in the PVN of the hypothalamus; these cells were also positive for Ucn1, indicating that VacA activated Ucn1-positive neurons in the PVN. Ucn1 is a 40-amino-acid peptide belonging to the CRF peptide family that exerts anorexigenic and anxiogenic effects^34^. Previous studies reported the distribution of Ucn1 in the brain including hypothalamus, Edinger–Westphal nucleus^35^, and the median eminence^36^ and the strain dependency in mice^37^. More recently, it has been shown that Ucn1 neurons in Edinger–Westphal nucleus are also involved in the control of food intake and energy metabolism^35^. Therefore, VacA might have affected entire brain including hypothalamus and Edinger-Westphal nucleus.

The inhibitory activity of Ucn1 on food intake is the most potent among the CRF family peptides (CRF and Ucn1, 2, and 3) in lean and high-fat diet-fed mice^38^. Central Ucn1 contributes to not only food intake but also gastrointestinal function, such as the suppression of gastric emptying and visceral pain^39,40^. These alterations induced by Ucn1 are similar to the symptoms observed in patients with *Hp*-associated FD^41^. Ucn1 binds with high affinity to both the CRF1 and 2 receptors^37^. The CRF1 receptors activate the hypothalamic-pituitary-adrenocortical (HPA) axis and cause anxiety-like behaviors in response to stress, whereas the CRF2 receptors mediate the inhibition of food intake^37,42,43^. We demonstrated that a CRF2 receptor antagonist inhibited the anorexigenic effect of VacA and that a CRF1 receptor antagonist ameliorated the VacA-induced anxiety-like behaviors induced by VacA in mice. Therefore, the Ucn1-CRF receptor axis might be an important mediator of the anorexigenic and anxiogenic effects of VacA.

VacA is thought to exert its effects by binding to receptors on various cells. LRP1 is a VacA receptor^23^. LRP1 expressed on gastric epithelial cells mediates vacuolation, autophagy, and apoptosis through the mitochondrial damage induced by VacA^23^. Conditional lrp1 forebrain KO mice have been reported to have higher body weights and food intake than were found in control lentivirus-injected mice, suggesting the contribution of LRP1 to the loss of body weight and food intake via the leptin signaling^24^. Leptin can directly influence the activity of the Ucn1 neurons^44^. Endogenous tissue-type plasminogen activator (tPA) is a perivascular ligand for LRP1 that increases permeability of the blood-brain barrier (BBB); an LRP1 antagonist blocks tPA-induced permeability of the BBB, suggesting that LRP1 might regulate BBB permeability^45^. Our studies strongly indicate that VacA secreted into peripheral blood infiltrates the BBB and can directly activate PVN neurons. The decrease in food intake and body weight and the increase in anxiety induced by VacA might be caused by the interactions between VacA in the PVN via the humoral pathway and hypothalamic LRP1.

Ghrelin is a peripheral orexigenic peptide that activates NPY/AgRP neurons in the hypothalamus^10^. The association with ghrelin and *Hp*-induced appetite loss remains a controversial issue. Our studies revealed that VacA has an inhibitory effect on food intake in ghrelin-KO mice and that ghrelin is not involved in the mechanisms of action of VacA.

The interaction between VacA and intracellular signaling has been demonstrated in epithelial and immune cells, including T cells^46^. In the present study, we used A7r5 cells to assess whether VacA activated intracellular signaling using calcium influx. Calcium influx is a marker of cell activation, such as protein secretion, cell proliferation and differentiation, and exocytosis, which is regulated by the PLC-PKC pathway^47^. VacA increased calcium influx in A7r5 cells, and VacA-induced calcium influx was blocked by a PLC inhibitor, a PKC inhibitor, and an anti-VacA antibody. These results suggest that VacA induces cell activation via the PLC-PKC pathway.

**Figure 6:**
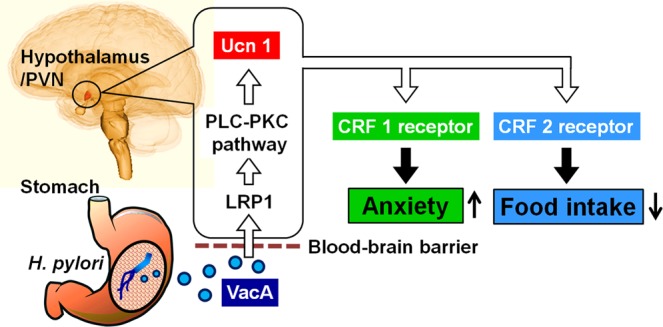
Schematic of the mechanism by which the VacA-Ucn1-CRF receptor axis induces anorexigenic and anxiogenic effects in response to *Hp* infection.

This is the first study demonstrating the anorexigenic and anxiogenic effects of VacA. We propose the following. VacA secreted by *Hp* in the stomach travels via the peripheral circulation and passes through the BBB. VacA binds to LRP1 and activates the intracellular PLC-PKC pathway, resulting in the activation of Ucn1-positive neurons, such as in the PVN of the hypothalamus. Secreted Ucn1 induces the inhibition of food intake through CRF2 receptors and anxiety through CRF1 receptors (Fig. 6). The central Ucn1-CRF receptor axis activated by VacA might be a new important pathway that contributes to the anorexigenic and anxiogenic effects of *Hp* infection and could be a therapeutic target for *Hp*-induced alterations.

## Methods

### Purification and activation of VacA

VacA was purified from a culture of the toxin-producing *Hp* strain ATCC 49503 (*s1m1* strain, American Type Culture Collection) according to a previous study^48^. In brief, after growth of the toxin-producing *Hp* in Brucella broth (Becton-Dickinson) containing 0.1% β-cyclodextrin (Sigma-Aldrich) at 37 °C for 3 to 4 days with vigorous shaking in a controlled microaerobic atmosphere of 10% O_2_ and 10% CO_2_, VacA was precipitated from the culture supernatant with a 50% saturation of ammonium sulfate. The precipitated proteins were dialyzed against dialysis buffer (RX buffer (mM): 10 KCl, 0.3 NaCl, 0.35 MgCl_2_, 0.125 ethylene glycol tetraacetic acid (EGTA), and 1 2-[4-(2-hydroxyethyl)-1-piperazinyl]ethanesulfonic acid (HEPES), *pH* 7.3) and were applied to an anti-VacA-specific immunoglobulin G (IgG) antibody column equilibrated with RX buffer. After the column was washed with RX buffer, VacA was eluted with 50 mM glycine-HCl buffer (*pH* 1.0), which was promptly neutralized with 1 M Tris (*pH* 10). After gel filtration on a Superose 6HR 10/30 column equilibrated with Tris-buffered saline (TBS: 60 mM Tris-HCl buffer, *pH* 7.7, containing 0.1 M NaCl), the purified VacA was concentrated (200 µg/ml) and stored at -20 °C. The VacA concentration was determined using a bead enzyme-linked immunosorbent assay method^49^. The vacuolating activity of purified VacA was verified according to a previous study^50^.

In all experiments, 0.1 M HCl (final concentration) was added to the purified VacA solution, which was then incubated at room temperature for 10 min. Then, 0.1 M NaOH (final concentration) was added to the VacA solution to neutralize the *pH* immediately prior to VacA administration.

### Animal treatments

Mongolian gerbils at 7 weeks of age and 45–50 g bw provided by Seac Yoshitomi (Fukuoka, Japan) were used. Male C57BL/6 J mice at 7 weeks of age and 20–25 g bw and male Wistar rats at 8 weeks of age and 200–250 g bw were purchased from CLEA Japan Inc. (Tokyo, Japan), and male Sprague-Dawley rats at 7 weeks of age and 200–250 g bw were purchased from SLC Japan, Inc. (Shizuoka, Japan). Male ghrelin-KO mice (C57BL/6 background) at 12–14 weeks of age and 20–25 g bw were provided by Professor Masayasu Kojima (Kurume University, Fukuoka, Japan)^51^.

Mongolian gerbils were maintained individually in a specific-pathogen-free (SPF) clean room under standard conditions at 24 ± 2 °C, 50 ± 10% humidity with a 12-h/12-h light-dark cycle and ad libitum access to sterile standard chow in the animal facility of Welfide Corporation. Mice and rats were maintained individually in an SPF clean room under standard conditions at 24 ± 2 °C, 50 ± 10% humidity with a 12-h/12-h light-dark cycle and ad libitum access to sterile standard chow (3.4 kcal/g; CE-2, CLEA Japan Inc., Tokyo, Japan) and water in the animal facility of Kagoshima university.

### *Hp* infection of Mongolian gerbils

The Mongolian gerbils were randomly assigned to the control group (*n* = 10) or the infection group (*n* = 10). After the animals were subjected to a 24-h fast, the infection group was orally inoculated with 1.6 × 10^7^ colony-forming units of *Hp* (ATCC 43504, *s1m1* strain, American Type Culture Collection, Rockville, MD) per animal, and the control animals were orally inoculated with Brucella broth (Becton-Dickinson, Cockeysville, MD), which was the reagent used for the cultivation of *Hp* in accordance with the modification described in a previous study^52^. Stomach tissues were isolated from the animals at the end of the experiments and were homogenized in phosphate-buffered saline (PBS) to confirm the presence of infection. Aliquots were diluted 5-fold with PBS and spread onto Helicobacter agar plates (Becton-Dickinson, Cockeysville, MD) containing 6.3% horse blood (Nihon Bio-test Laboratories Inc., Saitama, Japan), 2 µg/ml amphotericin (Sigma-Aldrich, St. Louis, MO), 10 µg/ml vancomycin (Sigma-Aldrich), 2.5 IU/ml polymyxin B (Sigma-Aldrich), 5 µg/ml trimethoprim (Sigma-Aldrich), and 50 µg/ml 2,3,5-triphenyl tetrazolium chloride (NacalaiTesque, Kyoto, Japan). The plates were incubated at 37 °C for 7 days before the colonies were counted.

### Cannula implantation for ICV administration of VacA

A sterile brain infusion cannula (28 gauge; Alzet; Durect Corp., Cupertino, CA) was implanted for chronic administration, while a guide cannula (25 gauge; Eicom, Kyoto, Japan) was implanted for acute administration. These cannulae reached the right lateral ventricle. Stereotaxic coordinates were established at 0.5 mm posterior to the bregma, 1.0 mm right lateral to the midline, and 2.5 mm below the outer surface of the skull using a Kopf stereotaxic frame (David Kopf Instruments, Tujunga, CA). For chronic administration, the infusion cannula was attached to the skull with dental cement, and the cannula was connected via polyvinylchloride tubing to an osmotic minipump (Alzet model no. 2004, Durect Corp., Cupertino, CA) filled with the VacA solution or artificial cerebrospinal fluid (ACSF (mM): 138.9 NaCl, 3.4 KCl, 1.26 CaCl_2_·2H_2_O, 4.0 NaHCO_3_, 0.6 NaH_2_PO_4_·2H_2_O, and 5.6 glucose). The pump was implanted under the skin of the back, and 50 mg/kg bw of antibiotics (Cefamezin, Astellas Pharma Inc., Tokyo, Japan) was administered subcutaneously. For acute administration, a guide cannula was implanted and attached with dental cement 7 days before the experiments. A dye solution (0.5% Evans blue and 5% Zelatin) was injected immediately after euthanasia, and histological examinations were performed on frozen brain sections to confirm the location of the cannula tip.

### Drug administration *in vivo*

Drug administration began between 8:30 and 9:30 a.m. VacA at doses of 3 and 30 nmol/kg bw in 100 µl of Tris-buffered saline (TBS) (60 mM Tris-HCl buffer, *pH* 7.7, containing 0.1 M NaCl) or TBS alone as the vehicle was IP administered to mice. VacA at doses of 0.06, 0.18, and 0.6 pmol/kg bw in 2 µl of ACSF or ACSF alone as the vehicle was administered via the ICV route to mice. To assess the contribution of the CRF receptors to VacA-induced behaviors, the mice were administered the CRF2 receptor antagonist antisauvagine-30 at a dose of 120 nmol/kg bw (Tocris Bioscience, Abingdon, UK) in 2 µl of ACSF, the CRF1 receptor antagonist NBI27914 at a dose of 400 nmol/kg bw (Tocris Bioscience) in 2 µl of a mixture of 80% dimethyl sulfoxide and 20% ACSF^53^, or each vehicle control alone via the ICV route 5 min after the ICV administration of VacA. In chronic ICV-administered male Sprague-Dawley rats, VacA in ACSF was infused via the ICV route at a dose of 0.12 pmol/day.

### Food intake and body weight measurements

In *Hp*-infected Mongolian gerbils, food intake and body weight were measured every 6 days for 198 days after inoculation. In the C57BL/6 J mice receiving the ICV infusion of VacA, cumulative food intake and body weight were measured every day for 5 days. In the experiments involving acute VacA administration, the C57BL/6 J and ghrelin-KO mice were fasted overnight with free access to water. The cumulative food intake was measured at 1, 2, 4, 8, 12, and 24 h after IP and ICV administration of VacA. In the mice receiving ICV administration of VacA and CRF receptor antagonists, cumulative food intake was measured for 8 h after administration. Sterile standard chow was used.

### Measurement of anxiety-like behaviors

Anxiety-like behaviors were measured 4 h after the administration of VacA with the elevated plus maze test as described in previous studies^54^. The mice were not exposed to overnight fasting before the elevated plus maze test. The mice were allowed to freely explore the maze for 5 min. We measured the time spent in the open arms, the closed arms, and the center area on the maze using a video tracking system (PANLAB ANART V2.0, Panlab Harvard Apparatus, Holliston, MA). We calculated the percentage of time spent in the open arms using the following formula: time spent in the open arms/(time spent in the open arms + time spent in the closed arms) × 100.

### Quantitative real-time polymerase chain reaction (qRT-PCR) analysis

The mice were perfused with 0.1 M phosphate buffer 4 h after IP VacA administration. Total RNA was extracted from isolated hypothalamus tissues using an RNeasy Plus Mini Kit (74134; QIAGEN, Hilden, Germany) after VacA administration, and cDNA was synthesized using a SuperScript III First-Strand Synthesis System (18080-051; Invitrogen, Carlsbad, CA) according to the manufacturer’s protocol. The qRT-PCR analysis of peptides was performed with SYBR Green Master Mix (Roche Inc., Basel, Switzerland) according to the manufacturer’s protocol. Relative mRNA levels were quantified using 2^−ΔΔCT^ method. Changes in mRNA expression were defined as significant if the 2^−ΔΔCT^ value increased more than 2-fold or decreased less than 0.5-fold. The primers used in the qRT-PCR are shown in Table S1. Ct values of GAPDH in hypothalamus samples are shown in Fig. S1.

### Immunohistochemistry

The mice were perfused with 4% paraformaldehyde and 0.5% glutaraldehyde in 0.1 M phosphate buffer 4 h after IP VacA administration. Brain sections (20 µm) of the NTS were incubated with a primary antibody against c-Fos (ABE 475, Merck Millipore, Belize, MA, 1:1000) for 48 h at 4 °C, incubated with a biotinylated anti-rabbit IgG solution (1:1,000) for 4 h at room temperature, visualized with the Vectastain ABC regents (Vector Laboratories, Burlingame, CA), and observed using a light microscope (Olympus DX51; Olympus Optical Co. Ltd., Tokyo, Japan). Hypothalamus sections were incubated with a primary antibody against c-Fos (sc-52-G, Santa Cruz Biotechnology, Inc., Dallas, TX, 1:1,000) and anti-PGP9.5 (GP14104, Neuromics, Edina, MN, 1:1,000) and/or urocortin1 (Ucn1, rabbit serum, Y362, Yanaihara Inc., Shizuoka, Japan, 1:100) or VacA (a generous gift from Dr. Hisao Kurazono, 1:1,000) for 48 h at 4 °C and then with a secondary antibody (Alexa Fluor 488-conjugated anti-guinea pig IgG, Jackson ImmunoResearch Laboratories Inc., West Grove, PA, 1:200, Alexa Fluor 555-conjugated anti-rabbit IgG, Abcam, Cambridge, MA, 1:200, and/or Alexa Fluor 647-conjugated anti-goat IgG, Jackson ImmunoResearch Laboratories Inc., 1:500) for 4 h at room temperature. Hypothalamus sections were observed using a confocal laser scanning microscope (LSM TCS SP8, Leica Microsystems, Wetzlar, Germany). Nuclei were counterstained with a 4′,6-diamidino-2-phenylindole dihydrochloride solution (DAPI, D523; Dojindo Molecular Technologies, Inc., Kumamoto, Japan). The antibodies used in the immunohistochemistry are shown in Table S2. Specificity of Ucn1 antibody and absence of its cross-reactivity with CRF has been previously described^55^.

### Measurement of [Ca^2+^]_i_ in single neurons isolated from the of rats

Brain sections containing the PVN were removed from Wistar rats. The dissected brain tissues were incubated with HEPES-buffered Krebs-Ringer bicarbonate buffer solution (HKRB (mM): 29 NaCl, 5.0 NaHCO_3_, 4.7 KCl, 1.2 KH_2_PO_4_, 2.0 CaCl_2_, 1.2 MgSO_4_, and 10.0 HEPES at *pH* 7.4) containing 1 mM glucose and incubated in 20 U/ml papain, 0.015 mg/ml deoxyribonuclease, 0.75 mg/ml bovine serum albumin, and 1 mM cysteine in HKRB for 15 min at 36 °C with shaking. The cell suspension was centrifuged at 100 × g for 5 min. The pellet was resuspended in HKRB and distributed onto coverslips. The cells were kept at 20 °C in moisture-saturated dishes for 30 min. The [Ca^2+^]_i_ in single cells was measured by ratiometric Fura-2 microfluorometry. Fura-2-acetoxymethyl ester (AM) is a membrane-permeable intracellular calcium indicator. When the molecule is cleaved by an intracellular esterase, the resulting Fura-2 exhibits sensitivity to Ca^2+^. Fluorescence images were detected every 8 s with an intensified charge-coupled device camera. Data were collected from cells identified as neurons by immunostaining for the neuron-specific marker microtubule-associated protein 2 according to a previously reported procedure^56^. The ratio image was produced by the Aquacosmos system (Hamamatsu Photonics Co., Hamamatsu, Japan). The activity of a single cell was validated by the [Ca^2+^]_i_ response to 30 mM KCl, which was tested at the end of each measurement.

### Measurement of [Ca^2+^]_i_ in cell models

We confirmed the expression of LRP1 by Western blotting (data not shown). The changes in [Ca^2+^]_i_ were detected with a fluorescent probe (Fura-2-AM, Dojindo Molecular Technologies, Inc.). Measurement of [Ca^2+^]i in A7r5 cells was conducted according to a previously reported procedure with slight modifications^57^. The cultured cells were placed in a buffered physiological saline solution (PSS (mM): 140 NaCl, 5.9 KCl, 1.2 CaCl_2_, 1.4 MgCl_2_, 11.5 glucose, and 1.8 Na_2_HPO_4_ in 10 HEPES-Tris buffer). Fura2-AM (F015, Dojindo Molecular Technologies, Inc.) was added to the solution at a final concentration of 5 μM, and the solution was incubated in a humidified 5% CO2/air atmosphere at 37 °C for 30 min. The cells were washed three times and resuspended in 900 μl of PSS, and 300 μl was loaded into the cuvette. The fluorescence was continuously recorded using a fluorescence spectrofluorometer (F-2000, Hitachi High-Technologies, Tokyo, Japan). The maximum fluorescence ratio (Rmax) was obtained by exposing cells to 10 mM of ionomycin, a Ca^2+^ ionophore, in PSS solution. Immediately after the Rmax was determined, the solution was replaced with Ca^2+^-free PSS solution containing 1 mM ethylene glycol-bis(β-aminoethyl ether)-*N,N,N’,N’*-tetraacetic acid (EGTA), and the minimum fluorescence ratio (Rmin) was determined. [Ca^2+^]i was calculated using the following formula: [Ca^2+^]i (nM) = (R−Rmin)/Rmax−R) × Kd × F380max/F380min, where R represents the fluorescence ratio (fluorescence intensity at excitation 340 nm/fluorescence intensity at excitation 380 nm), Rmin represents R in the absence of calcium by exposure to 1 mM EGTA, Rmax represents R in the presence of a saturating concentration of calcium, Kd represents the dissociation constant for the dissociation of calcium from Fura-2 (225 nM), and F380max and F380min represents the ratio of the baseline fluorescence (380 nm) under calcium-free and calcium-bound conditions, respectively. After the baseline value was recorded, VacA (0.03, 0.3, and 3 μM) was added to the cuvette, and [Ca^2+^]i was measured following a 30-min pretreatment with the phospholipase C (PLC) inhibitor U-73122 (0.01, 0.1, and 1 nM, Tocris Bioscience), the protein kinase C (PKC) inhibitor chelerythrine (0.01, 0.1, and 1 nM, Tocris Bioscience), or anti-VacA polyclonal antibodies (a generous gift from Dr. Hisao Kurazono, 1:1,000, 1:500, and 1:250).

### Data analysis

The data are presented as the means ± standard errors of the mean (SEM). Comparisons between two groups were performed using two-tailed Student’s *t*-tests and Mann-Whitney tests. One-way or two-way analysis of variance (ANOVA) followed by Tukey’s multiple comparison test was used to compare three or more groups. Differences were considered significant at *p* < 0.05. All statistical analyses were performed using Prism 6 software (GraphPad, San Diego, CA).

### Ethics Approval

The animal protocols for this study were approved by the Kagoshima University Committee for Animal Experiments, the Jichi Medical University Institute of Animal Care and Use Committee, and the Institutional Animal Care and Use Committee of Welfide Corporation (Osaka, Japan). All experiments were performed in accordance with relevant guidelines and regulations.

## Supplementary information

Supplementary Information
