# Supplementary material for: Helicobacter pylori Vacuolating Cytotoxin A Causes Anorexia and Anxiety via Hypothalamic Urocortin 1 in Mice
Source: Sci Rep. 2019 Apr 12;9:6011. doi: 10.1038/s41598-019-42163-4 (PMC6461611; doi:10.1038/s41598-019-42163-4)
Supplement: Supplementary file 1 — Supplementary Information [file 41598_2019_42163_MOESM1_ESM.pdf]

Supplementary Information

Helicobacter pylori Vacuolating Cytotoxin A Causes Anorexia and Anxiety via Hypothalamic Urocortin 1 in Mice

Hajime Suzuki, Koji Ataka, Akihiro Asakawa, Kai-Chun Cheng, Miharu Ushikai, Haruki Iwai, Takakazu Yagi, Takeshi Arai, Kinnosuke Yahiro, Katsuhiro Yamamoto, Yoshito Yokoyama, Masayasu Kojima, Toshihiko Yada, Toshiya Hirayama, Norifumi Nakamura, and Akio Inui

Figure legend

Fig. S1. Ct values of GAPDH in hypothalamus samples. There were no significant differences in Ct values of GAPDH in our experiments. The expressions of GAPDH were stable in these hypothalamus samples.

Fig.S1

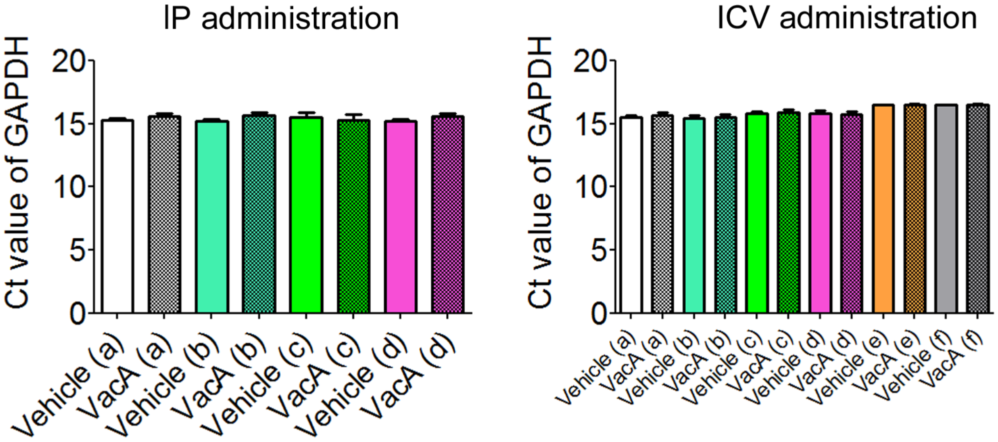

a: AgRP. NPY. CRF, POMC, CART

b: OXT, Ucn1, Ucn2, Ucn3, AVP

c: Orexin, MCH

d: LRP1

a: AgRP, NPY, CRF

b: OXT, AVP, LRP1

c: POMC, CART, Ucn3

d: Ucn1, Ucn2

e: Orexin

f: MCH

Supplementary Table 1

		Forward	Reverse
agouti-related peptide (AgRP)	NM_001271806	GCGGCCTGAAAGCTTTGTC	TCCTGTAGCCAGGGCATGAG
neuropeptide Y (NPY)	NM_023456	CGCTCTGCGACACTACATCAAT	TGAGATGAGGGTGGAAACTTGG
melanin-concentrating hormone (MCH)	NM_029971	AAACGACGAGAGCGGTTTCA	TTCCTGTGTGGACTCAGCATTC
orexin	NM_010410.2	CGTAACTACCACCGCTTTAGCA	TGCCATTTACCAAGAGACTGACA
proopiomelanocortin (POMC)	NM_001278581	AGAGGCCACTGAACATCTTTGTC	TCTATGGAGGTCTGAAGCAGGAG
cocaine- and amphetamine-regulated transcript (CART)	NM_013732	TCAAGAGTAAACGCATTCCGATCTA	TCCTCACTGCGCACTGCTCT
corticotropin-releasing factor (CRF)	NM_205769	CAGAGCCCAAGTACGTTGAGAG	GCTCTCTTCTCCTCCCTTGGTA

urocortin1 (Ucn1)	NM_021290	CATCTTGCACTGGGCAGACACT	AAGCTGTGCCAAGAGCAGCAAC
Ucn2	NM_145077	ATGTTTGGGCATCTTGGACTCT	GCCTGTGGACCTTAGATGGACT
Ucn3	NM_031250	ACCAGAGAAGACAAAGCTGCAA	GAAGTGGCAGCAGGAAGTAGGT
oxytocin (OXT)	NM_011025	TGCCAGGAGGAGAACTACCTG	TATTCCCAGAAAGTGGGCTCAG
arginine vasopressin (AVP)	NM_009732	TCTCTGACATGGAGCTGAGACAG	AGGGCAGGTAGTTCTCCTCCT
low-density lipoprotein receptor-related protein-1 (LRP1)	NM_008512	TCGGCAGACCATCATCCAAG	ATTGTCCGAGTTGGTGGCGTA.
glyceraldehyde-3-phosphate dehydrogenase (GAPDH)	NM_008084	CTACAGCAACAGGGTGGTGGAC	GGATAGGGCCTCTCTTGCTCAG

Table S2

Primary antibody	Secondary antibody
NTS sections	
c-Fos (ABE 475, Merck Millipore, Billerica, MA, 1:1,000)	Biotinylated anti-rabbit IgG (Vector Laboratories, Burlingame, CA, 1:200)
Hypothalamus sections	
c-Fos (sc-52-G, Santa Cruz Biotechnology, Dallas, TX, 1:1,000)	Alexa Fluor 647-conjugated anti-goat IgG (Jackson ImmunoResearch Laboratories Inc., West Grove, PA, 1:500)
Urocortin1, rabbit serum (Y362, Yanaihara Inc., Shizuoka, Japan, 1:100)	Alexa Fluor 555-conjugated ant-rabbit IgG (Abcam, Cambridge, MA, 1:200)
VacA (a generous gift from Dr. Hisao Kurazono, 1:1,000)	Alexa Fluor 555-conjugated ant-rabbit IgG (Abcam, Cambridge, MA, 1:500)
PGP9.5 (GP14104, Neuromics, Edina, MN, 1:1,000)	Alexa Fluor 488-conjugated anti-guinea pig IgG (Jackson ImmunoResearch Laboratories Inc., West Grove, PA, 1:200)
